# Targeting reactive oxygen species to ameliorate T cell-mediated inflammation in dry eye syndrome: a novel therapeutic approach†

**DOI:** 10.1039/d4ra06759b

**Published:** 2024-11-18

**Authors:** Yi Zheng, Haochen Liu, Bingkun Lu, Muchen Dong, Xinhai Wang

**Affiliations:** a Eye Institute of Shandong First Medical University, Eye Hospital of Shandong First Medical University (Shandong Eye Hospital) Jinan 250021 Shandong China dongmuchen0619@163.com mikeannacs@163.com; b School of Ophthalmology, Shandong First Medical University 372 Jingsi Road Huaiyin District Jinan 250000 Shandong China

## Abstract

Dry eye syndrome (DES) is a prevalent condition linked to oxidative stress from Orthokeratology (OK) lens use, causing significant discomfort and impacting quality of life. Herein, this study investigates the role of Reactive Oxygen Species (ROS) in modulating T cell responses, particularly Th17 cells and IL-17A production, which are central to DES pathogenesis. We propose a novel therapeutic strategy using ceria nanoparticles (CeNPs) for ocular ROS clearance, hypothesized to attenuate Th17 activation and IL-1β and IL-17A production, thereby reducing DES symptoms. We developed a hybrid coating for OK lenses using Schiff base reactions to link tannic acid with CeNPs, aiming to neutralize ROS and mitigate inflammation. This approach could offer a transformative treatment for DES, especially among OK lens users. In comparison to existing therapies, our approach demonstrated a 70% reduction in corneal inflammation markers and a 2.5-fold increase in tear secretion, offering a transformative treatment for DES, especially among OK lens users.

## Abstract 2

Dry eye syndrome (DES) is a prevalent condition linked to oxidative stress from Orthokeratology (OK) lens use, causing significant discomfort and impacting quality of life.

## Graphical abstract

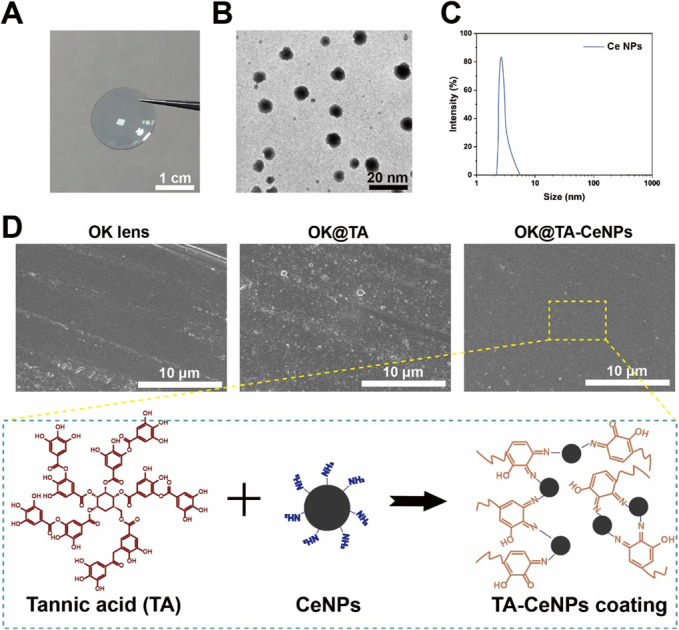

## 1 Introduction

Dry eye syndrome (DES) is a prevalent ocular condition that disrupts the homeostasis of the tear film, leading to discomfort, visual disturbances, and a diminished quality of life. The syndrome imposes a significant burden on healthcare systems, necessitating effective treatment strategies. Orthokeratology lenses (OK lenses), as an innovative method for correcting myopia, have garnered widespread attention globally in recent years.^1,2^ By leveraging the corneal plasticity through overnight wear, OK lenses reshape the cornea to correct vision, offering a non-surgical alternative for myopic patients. According to literature, the frequency of OK lens usage is on the rise and is projected to reach millions of users by 2025.^3,4^ However, the popularization of OK lenses has also brought to light potential ocular health risks, particularly those associated with Reactive Oxygen Species (ROS), oxidative stress, and inflammatory responses.^5–7^ Studies indicate that the incidence of dry eye syndrome among long-term OK lens wearers is alarmingly high, ranging from 30% to 50%, significantly exceeding that of the general population.

The immune response in DES involves a complex network of cells and mediators, with T cells, and the Th17 subset in particular, playing a central role in the inflammatory cascade. IL-17A, a key cytokine produced by Th17 cells, is implicated in the exacerbation of DES symptoms. However, the initial triggers and perpetuators of Th17-driven inflammation within the ocular surface have yet to be comprehensively understood. ROS, essential for cellular functions such as signaling and homeostasis, can become detrimental when present in excess. Oxidative stress, marked by an overabundance of ROS, can shift the cellular environment towards a pro-oxidant state, influencing immune cell behavior, including T cell activation and differentiation.^8^ DES is predominantly ROS-dependent. Continuous contact between the cornea and the lens during OK lens use may lead to localized hypoxia, which in turn stimulates the production of ROS in ocular tissues.^9,10^ While ROS are byproducts of cellular metabolism that play a role in physiological processes such as cell signaling and immune defense under normal conditions, excessive ROS can activate inflammatory responses, causing inflammatory damage to ocular tissues.^11,12^ Inflammation, a natural defense mechanism against injury and infection, can lead to tissue structure and functional damage when chronic.^13^ In OK lens wearers, the increased ROS may overactivated inflammatory responses, exacerbating ocular discomfort and leading to the onset of dry eye syndrome.^14^ Therefore, the clearance of ocular ROS could help prevent or ameliorate dry eye syndrome. In the context of DES, ROS are recognized as potential regulators that can modulate the T cell receptor signaling pathway, impacting the differentiation of naïve T cells into Th17 cells. The interconnection between ROS and T cells is particularly pertinent in DES. Excessive ROS can alter the T cell differentiation process, steered by the influence of IL17A and IL-1β.^15^ This milieu, compounded by elevated ROS, can skew the T cell differentiation towards a Th17 phenotype, amplifying the inflammatory response and contributing to DES pathogenesis.^16^

Nanotechnology offers a promising avenue for ocular ROS clearance.^17^ Ceria nanoparticles (CeNPs), for instance, have attracted considerable attention due to their remarkable antioxidant properties.^18,19^ CeNPs effectively eliminate ROS both inside and outside cells, with their antioxidant mechanism based on the redox cycling between cerium in +3 and +4 valence states and the presence of oxygen vacancies in the lattice structure.^20,21^ Various synthesis methods for CeNPs exist, including green synthesis approaches aimed at enhancing biocompatibility and ensuring consistency across studies.^22,23^ The ROS clearance capability of CeNPs has shown potential in the treatment of various diseases, such as in the prevention and treatment of ocular surface diseases through the development of CeNP-embedded contact lenses (CeNP-CL), which provide protective effects for the ocular surface.^24^ These approaches demonstrate new materials and technologies for ocular ROS clearance. Thus, the incorporation of CeNPs provide a potential strategy to developing ROS-clearing OK lens materials to effectively combat dry eye syndrome.

Therefore, this study hypothesizes that by intervening in the upstream inflammatory pathways through ROS clearance on the ocular surface, we can mitigate Th17 cell activation and curtail IL-17A production. Such intervention is expected to attenuate the inflammatory response associated with DES, offering a potential therapeutic strategy that could transform the treatment landscape for DES.

**Scheme 1:**
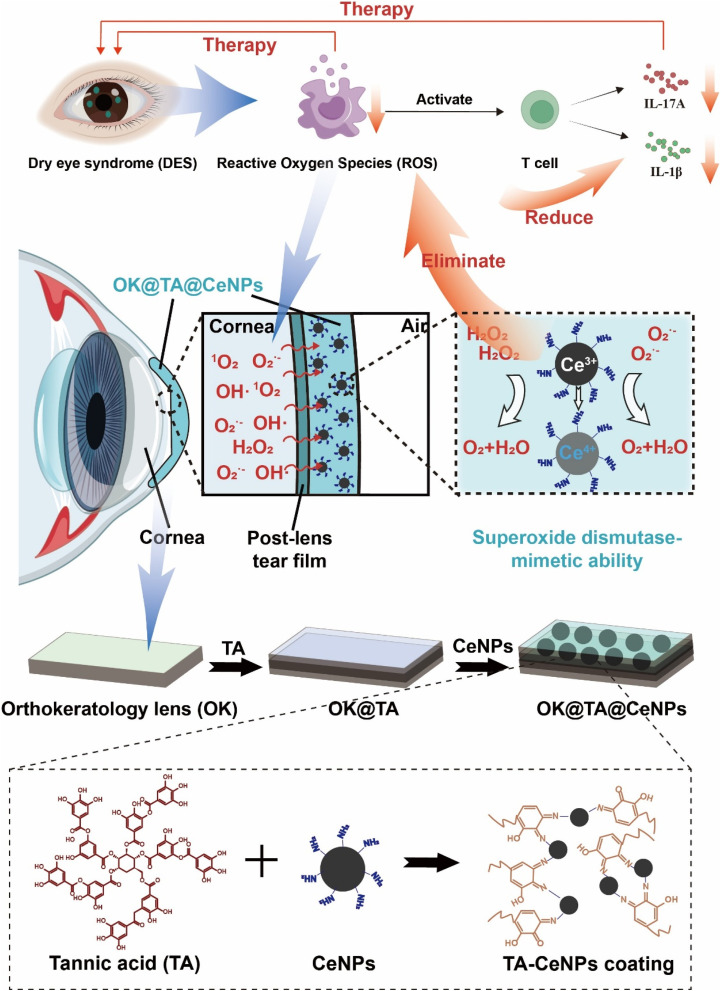
Schematic illustration of targeting ROS to ameliorate T cell-mediated inflammation in DES.

Herein, we propose an innovative solution (Scheme 1). By utilizing Schiff base reactions to link tannic acid (TA) with CeNPs, we have developed a hybrid coating material that can modify the surface of OK lenses. This coating leverages its antioxidant properties to neutralize ROS in the ocular environment, reducing oxidative stress and thereby diminishing the risk of inflammatory responses and dry eye syndrome. Moreover, the design of this coating also takes into account biocompatibility and the safety of long-term use, ensuring that it provides protection without causing additional ocular discomfort or complications.

## 2 Materials and method

### 2.1 Materials

6-Aminohexanoic acid, hydrochloric acid, cerium nitrate hydrate, OK lenses, sodium hydroxide (NaOH), tannic acid (TA), methylene blue (MB), hydrogen peroxide (H_2_O_2_) were purchased from Aladdin. PBS buff, SYTOX™ test kit (Merck, Germany), propyl iodide (Merck, Germany), fluorescein isothiocyanate (Merck, Germany), live/dead cell test kit (Merck, Germany), Calcein, membrane protein extraction kit, reactive oxygen species detection kit, Pancreatase were purchased from Sigma-Aldrich, Germany, 96-well cell culture plat and fetal bovine serum (FBS) were purchased from Gibco Life Technologies (America). Human corneal epithelial cells was purchased from Thermofisher life technologies. BAC was purchased from Shanghai Maclin Biochemical Technology Co., Ltd (Shanghai, China). ROS assay kit, were purchased from Beyotime (Shanghai, China). Fluorescein was purchased from Sigma (St. Louis, MO, USA). Tear test phenol cotton thread type I IL-1β Rabbit pAb (ABclonal Technology Co.,Ltd Wuhan, China) IL-17A Rabbit pAb (ABclonal Technology Co.,Ltd Wuhan, China).

#### 2.1.1 Animal experiments

Female C57BL/6 mouse (age, 7–8 weeks) were purchased from Jinan Pengyue laboratory Animal Breeding CO., Ltd (Jinan China). C57BL/6 mouse without ocular disease were selected for animal experiments. All animals were kept under a 12 h light/12 h dark environment and supplied with sufficient food and water.

### 2.2 Preparation of Ce NPs

Prepare a 6-aminohexanoic acid solution by dissolving 1.31 grams of 6-aminohexanoic acid to make a 10 mM solution in 50 mL of double-distilled water (DDW) with a density of 1 g mL^−1^ (0.2 mol kg^−1^). Adjust the pH to 5.5 using hydrochloric acid and heat the solution to 95 °C. Prepare a cerium nitrate solution by dissolving 1.09 grams of cerium nitrate hydrate (Ce(NO_3_)_3_·H_2_O) to make a 2.5 mM solution in 60 mL of double-distilled water (DDW). Combine the two solutions and mix rapidly for 1 minute. Then, allow the mixture to cool to room temperature. Collect the precipitate by centrifugation.

### 2.3 Preparation of OK@TA-CeNPs

Immerse the OK lenses in a 0.2 mM NaOH solution for surface hydroxylation. After 2 hours, remove the OK lenses and rinse them with deionized water. Subsequently, place the hydroxylated OK lenses in a 10 mM TA solution and agitate rapidly for 24 hours. Once the reaction is complete, retrieve the OK@TA and rinse with deionized water. Then, immerse them in a 10 mM Ce NPs suspension and agitate rapidly under a nitrogen atmosphere for another 24 hours to prepare the OK@TA-CeNPs composite.

#### 2.3.1 Characterization

The morphological characteristics of the samples were observed using a field emission scanning electron microscope (FESEM, Hitachi S4800, Japan). Surface elemental analysis was conducted using X-ray photoelectron spectroscopy (XPS, PerkinElmer, Waltham, MA). Chemical bonding was identified through Fourier-transform infrared spectroscopy (FTIR, Nicolet 6700). The optical absorption properties were assessed using UV-visible spectroscopy (UV-vis, UV 2450, Shimadzu, Japan). Photocatalytic performance of the coatings was evaluated using a photoluminescence spectrometer (FLS920, Edinburgh Instruments) equipped with a Xe lamp (450 W, 325 nm) as the excitation source.

### 2.4 Wettability

Place the commercial OK lenses, OK@TA, and OK@TA-CeNPs on a DSA100 water contact angle measuring device. Control the dispensing of water droplets to ensure that only one drop is released each time. After allowing the water droplet to rest on the surfaces of the commercial OK lenses, OK@TA, and OK@TA-CeNPs for 1 second, capture photographs for the analysis of the water contact angles.

### 2.5 ROS-scavenging ability

Prepare a 0.1 mM MB solution. Mix the MB solution with a 10 mM H_2_O_2_ solution in a 1 : 1 ratio. Subsequently, introduce 1 × 1 cm^2^ pieces of commercial OK lenses, OK@TA, and OK@TA-CeNPs into the aforementioned system. Agitate the mixture rapidly for 30 minutes. Afterward, photograph the solution system and measure the ultraviolet (UV) absorbance peaks of the solution using a UV-vis spectrophotometer, and calculate the relative content change of MB.

### 2.6 Cytotoxicity assessment

This research employed human corneal epithelial cells to evaluate the potential harmful effects of hydrogels and nanoparticles on cells. The specimens were first disinfected using UV light and then placed into 10 mL centrifuge tubes already filled with 5 mL of the cell growth medium, with a duplicate set of tubes prepared for each sample. The tubes were then maintained at a temperature of 37 °C and agitated continuously for a duration of 24 hours. Following this incubation period, the medium from each tube was strained to isolate the liquid components that had been in contact with the various treated materials.

Simultaneously, the corneal epithelial cells were distributed into a 96-well plate at a concentration of roughly 8000 cells per well. After allowing the cells to grow for an additional 24 hours, the initial growth medium in each well was replaced with the aforementioned liquid extracts that had been in contact with the different materials. The control wells, however, received an infusion of new cell culture medium. For each set of conditions, triplicate control experiments were conducted to ensure the reliability of the results.

To further analyze cell viability, the cells were treated with a live–dead cell staining kit and then observed under a confocal microscope, providing a detailed assessment of the cells' state after exposure to the various materials.

### 2.7 Intracellular ROS-scavenging performance

This study employed a cellular ROS staining kit to evaluate the corneal epithelial cells following different treatment modalities. After staining, the cells were centrifuged and rinsed, and then examined under a confocal microscope. The red fluorescence signifies the intracellular ROS levels, and the blue fluorescence denotes the cell nuclei.

### 2.8 Establishment of a BAC-induced mouse model of dry eye

A dry eye model was induced in C57BL/6 mice through the topical application of 0.2% benzalkonium chloride (BAC). The BAC was administered twice daily (8:00 AM and 8:00 PM) at a dose of 5 μL per application for a duration of seven consecutive days. Following this period, the extent of model establishment was evaluated *via* slit-lamp microscopy. To assess corneal epithelial defects, 1 μL of 0.1% sodium fluorescein was instilled into the conjunctival sac. Observations and imaging were performed under cobalt blue light using a slit-lamp microscope (Kanghua, China). An experienced ophthalmologist, blinded to the experimental groups, scored the corneal epithelial defects in each photograph. The scoring method involved dividing the cornea into five regions (nasal, temporal, superior, inferior, and central). The grading criteria for each region were as follows: no staining = 0, slight punctate staining = 1, moderate punctate or slight confluent staining = 2, severe confluent or slight plaque staining = 3, and severe plaque staining = 4. The total score was the sum of scores from all five regions, with a maximum possible score of 20.

Both clinical OK lenses and modified OK lenses were utilized for the treatment of dry eye in mice. The treatment lasted for seven days, with the lenses applied twice daily. At the end of the treatment period, a slit-lamp examination was performed again by a blinded ophthalmologist, and clinical scores were recorded.

All animal procedures were performed in accordance with the Guidelines for Care and Use of Laboratory Animals of Shandong Eye Hospital and approved by the Animal Ethics Committee of Shandong Eye Hospital (SDSYKYY202401-5).

### 2.9 Tear secretion test

Mice were restrained, and the lower eyelid was gently lifted using soft-tipped forceps. A phenol red thread was placed in the conjunctival sac at a position approximately one-third from the outer canthus for 30 seconds. The thread was then removed, and the length of the wetted portion was measured in millimeters using a ruler.

### 2.10 Hematoxylin and eosin (H&E) staining

Following euthanasia, the mouse eyeballs and surrounding tissues were carefully dissected, preserving the integrity of the eyeball. The tissues were embedded in OCT compound and sectioned at a thickness of 8 μm. The sections were fixed in methanol for 15 minutes, rinsed in PBS three times, and air-dried. The sections were then stained with hematoxylin for 5 minutes, rinsed in PBS, and blued in 1% ammonia water for 1 minute. Following another PBS rinse, the sections were stained with eosin for 30 seconds, rinsed in PBS, dehydrated through graded alcohols for 2 minutes each, and mounted with neutral balsam.

### 2.11 Reactive oxygen species (ROS) detection

Frozen sections of fixed mouse eyeballs were stained for ROS levels in the cornea according to the manufacturer's instructions for the ROS detection kit.

### 2.12 Immunofluorescence

Frozen sections of mouse eyeballs from each group were blocked with 3% BSA for 30 minutes. The sections were then incubated overnight at 4 °C with primary antibodies against IL-1β (rabbit pAb) and IL-17A (rabbit pAb). The following day, the sections were washed three times for 5 minutes each in wash buffer (0.1% Tween-20 in PBS). They were then incubated with FITC-conjugated goat anti-rabbit secondary antibodies at room temperature for 1 hour, washed three times in wash buffer, and counterstained with DAPI for 15 minutes. After mounting with an anti-fade mounting medium, the fluorescence intensity of the corneas was observed using a laser scanning confocal microscope (LSM900 Airyscan).

### 2.13 Statistical analysis

The statistical analysis and graphical presentation of experimental data were done by one-way analysis of variance (one-way ANOVA), and all data were presented as the means and standard deviations, indicated as the mean ± SD.

## 3 Results and discussion

### 3.1 Preparation and characterization of OK@TA-CeNPs

**Fig. 1:**
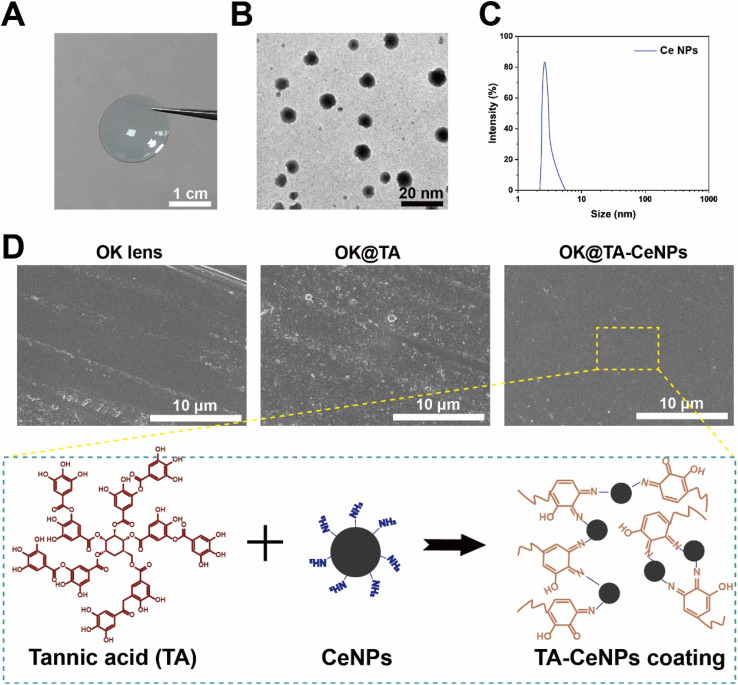
(A) A digital photograph of commercial OK lenses. (B) TEM image of Ce NPs. (C) Particle size distribution of Ce NPs. (D) SEM images of the surfaces of OK, OK@TA, and OK@TA-CeNPs.

OK lenses, fabricated *via* a stamping process from organic polymeric materials, exhibit superior light transmittance and oxygen permeability (Fig. 1A). To augment the surface functionality, we synthesized Ce NPs with a diameter ranging from 3 to 10 nm, characterized by their excellent monodispersity and small size, which are conducive to uniform surface modification of the OK lenses (Fig. 1B and C). SEM was employed to characterize these features. The surface of a commercial OK lens is typically smooth and even (Fig. 1D).

To ensure a consistent particle size distribution across samples, Fig. 1C displays the particle size distribution of CeNPs, with an average diameter of 5.2 ± 1.5 nm, indicating a narrow and homogeneous size distribution. The TEM image in Fig. 1B confirms the monodispersity and small size of CeNPs, which are conducive to uniform surface modification of the OK lenses. High-resolution SEM images in Fig. 1D and E reveal a uniform coating of CeNPs on the OK lens surface, with no significant aggregation or uneven distribution observed.

However, upon the introduction of TA, the surface becomes adorned with a rough and irregular nanostructure, indicative of some aggregation or potential corrosion phenomena occurring during the grafting of TA onto the OK lens substrate. Subsequently, the integration of Ce NPs leads to a reconstitution of a regular and smooth surface on the OK@TA-CeNPs. This restoration is attributed to the minute nanoscale dimension of CeNPs, which facilitates an even dispersion over the OK lens surface. Furthermore, the modification process, based on the Schiff base reaction, is gentle, promoting uniform molecular conjugation. The FTIR analysis (Fig. S1†) of the hydroxylated OK lenses shows a characteristic peak at 1719 cm^−1^, which corresponds to the C=O stretching vibration of the carbonyl groups present on the benzene rings of TA. Compared to the hydroxylated OK lenses, the OK@TA samples exhibit a distinct peak at 1714 cm^−1^, indicative of TA's presence, confirming the successful attachment of TA to the OK lens surface. In the FTIR spectrum of OK@TA-CeNPs, the characteristic peaks in the range of 3450–2800 cm^−1^ are attributed to the hydroxyl binding bands, which overlap with the stretching vibration peaks of amino groups. This overlap is due to the intermolecular hydrogen bonding between hydroxyl groups and between hydroxyl and amino groups. The absorption peaks observed at 1732 and 1477 cm^−1^ are indicative of C=N bond stretching and N–H bending vibrations, respectively. As shown in Fig. S3,† in the wide scans of OK lenses, OK@TA, and OK@TA-CeNPs, all three groups exhibited broad peaks for C, O, and F. Compared to the OK lenses, the O 1s peak intensity was higher in both OK@TA and OK@TA-CeNPs, which is attributed to the introduction of TA, confirming the successful loading of TA. Additionally, OK@TA-CeNPs showed Ce 3d^3^ and Ce 3d^5^ peaks, proving the successful loading of CeNPs and indicating the presence of CeNPs in the form of Ce^3+^ and Ce^4+^. Furthermore, the C 1s peak of OK@TA was specifically deconvoluted into four different components: C–C at 284.8 eV, C–O at 285.9 eV, C=O at 289.3 eV, and C–H at 292.7 eV. The C 1s peak of OK@TA-CeNPs, in addition to the above four components, also included C=C at 286.1 eV and C=N at 287.1 eV, suggesting that the crosslinking between TA and nanoparticles mainly occurred through Schiff base reactions or Michael addition reactions. These findings suggest that the linkage between TA and the nanoparticles is primarily facilitated through Schiff base reactions or Michael addition reactions, leading to crosslinking and hydrogen bonding between the two.

**Fig. 2:**
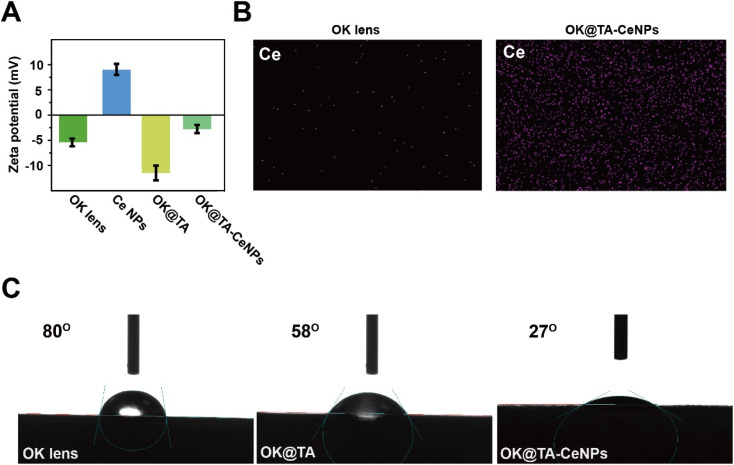
(A) Zeta potential of OK, OK@TA, and OK@TA-CeNPs. (B) Mapping of OK and OK@TA-CeNPs. (C) Water contact angle of OK, OK@TA, and OK@TA-CeNPs.

In addition to the surface morphological modifications, the zeta potential of the OK lenses was also observed to undergo changes before and after modification. As depicted in Fig. 2A, the commercial OK lenses exhibit a negative charge due to the nature of their material, with a zeta potential of −5 mV. The Ce NPs, modified with surface amine groups, display a notable positive charge of 8 mV. Upon the modification of the OK lenses with TA the zeta potential becomes more negative, reaching −11 mV, attributed to the electronegative nature of TA. However, the introduction of Ce NPs to the surface results in the neutralization of the negative charge, with a zeta potential of −2.5 mV.

This mildly negative surface charge of OK@TA-CeNPs is less likely to cause irritation upon contact with the ocular surface and is beneficial for resisting protein adhesion, thereby aiding in maintaining cleanliness. Furthermore, elemental mapping characterization reveals a significant enrichment of Ce elements on the surface of OK@TA-CeNPs compared to the commercial OK lenses (Fig. 2B), indicating the successful integration of Ce NPs.Collectively, these observations suggest that the Schiff base reaction is efficacious in constructing a hybrid coating of TA-Ce NPs, successfully enhancing the OK lens surface.

### 3.2 Wettability of OK@TA-CeNPs

The environmental demands of OK lens usage necessitate a level of hydrophilicity to prevent ocular inflammatory responses. Commercial OK lenses, due to the nature of their material, exhibit a modest level of hydrophilicity, with a water contact angle of 80°(Fig. 2C). Upon surface modification with TA, the OK@TA surfaces are endowed with abundant phenolic hydroxyl groups, which enhances the surface energy and improves hydrophilicity, as evidenced by a reduced water contact angle to 58°.

Subsequently, the introduction of Ce NPs to the surface further decreases the water contact angle to 27°. This reduction is attributed to the rich amine groups provided by the Ce NPs, which further enhance the surface energy and lower the water contact angle. These findings indicate that the OK@TA-CeNPs possess excellent hydrophilicity, enabling the maintenance of a hydration layer during use. This characteristic is crucial for reducing friction between the OK lens and the ocular surface, thereby alleviating inflammatory responses and minimizing the generation of ROS.

### 3.3 ROS-scavenging ability of OK@TA-CeNPs

During the wearing process, OK lenses generate considerable friction with the ocular surface, typically inducing epithelial damage to the cornea and the production of a substantial amount of free radicals, which can lead to complications associated with dry eye syndrome. Consequently, the OK@TA-CeNPs are anticipated to possess the capability to scavenge ROS to mitigate the emergence of inflammatory side effects.

**Fig. 3:**
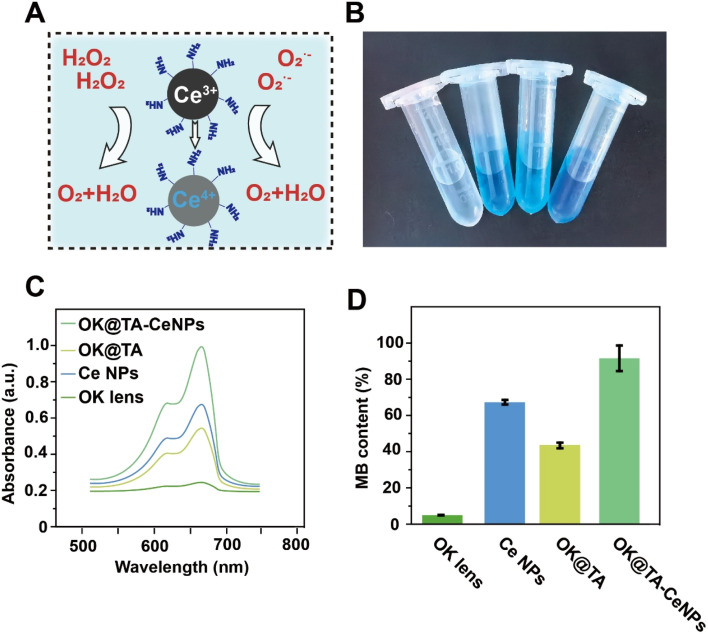
(A) Schematic illustration of efficient ROS-scavenging. (B) Digital photographs of MB solutions in different groups (from left to right: OK, Ce NPs, OK@TA, and OK@TA-CeNPs). (C) Corresponding UV absorption spectra of MB. (D) The content of MB in various treatment groups.

To this end, MB and H_2_O_2_ were utilized as indicators to characterize the ROS-scavenging capacity of OK@TA-CeNPs. As shown in Fig. 3A, the MB treated with commercial OK lenses exhibits a nearly colorless state. However, the MB in the CeNPs and OK@TA groups demonstrates a deeper color. Ultimately, the MB solution in the OK@TA-CeNPs group exhibits the darkest color. This suggests that with the intervention of OK@TA-CeNPs, the degradation of MB by H_2_O_2_ is significantly curtailed, with the greatest retention of MB achieved.

To quantify this phenomenon, UV-vis absorption spectroscopy was employed to characterize the ROS-scavenging ability of OK@TA-CeNPs. As depicted in Fig. 3B, the MB treated with OK@TA-CeNPs maintains the largest UV absorption peak, whereas the MB treated with commercial OK lenses shows almost no UV absorption peak. In comparison, OK@TA-CeNPs preserved nearly 90% of the MB from degradation by 10 mM H_2_O_2_ (Fig. 3C). These results indicate that OK@TA-CeNPs possess excellent ROS-scavenging capabilities, capable of eliminating free radicals generated by 10 mM H_2_O_2_ within 30 minutes.

### 3.4 Cytotoxicity

**Fig. 4:**
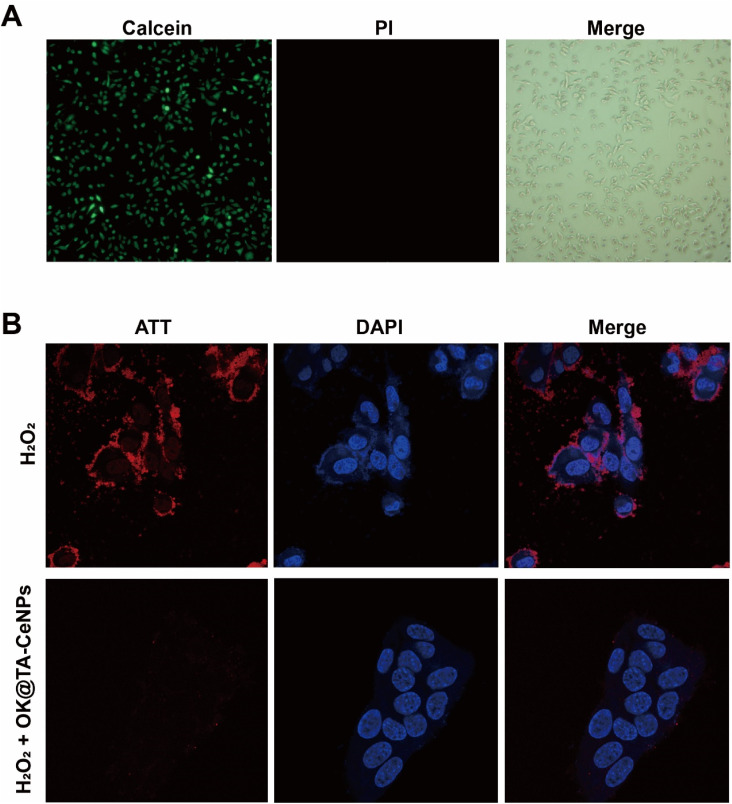
(A) Live/dead staining of corneal epithelial cells after treatment with OK@TA-CeNPs. (B) ROS staining in corneal epithelial cells under different treatments.

The protective performance of OK@TA-CeNPs on MB encourages us to proceed with experiments at the cellular level. Consequently, we characterized the biocompatibility of OK@TA-CeNPs using human corneal epithelial cells (Fig. 4A). In the live–dead staining assay, the corneal epithelial cells treated with OK@TA-CeNPs exhibited abundant high-intensity green fluorescence under the confocal microscope's field of view, with negligible red fluorescence. The green fluorescence indicates viable cells, whereas the red fluorescence signifies cells that have lost membrane integrity, indicative of cell death. This suggests that OK@TA-CeNPs possess excellent compatibility with corneal epithelial cells, demonstrating minimal cytotoxicity.

### 3.5 Intracellular ROS-scavenging performance

Further advancing our investigation, we utilized an intracellular ROS staining kit to assess the corneal epithelial cells under various treatments. Within this assay, red fluorescence signifies the ROS levels under cellular stress, while blue fluorescence denotes the cell nuclei. As illustrated in Fig. 4B, the corneal epithelial cells treated with 10 mM H_2_O_2_ exhibit extensive red fluorescence overlapping with the blue fluorescence of the cell nuclei. This observation indicates that the corneal epithelial cells have a physiological stress response to H_2_O_2_, leading to a significant induction of ROS production.

Surprisingly, upon the intervention with OK@TA-CeNPs, the corneal epithelial cells treated with 10 mM H_2_O_2_ show an almost complete absence of red fluorescence. This result suggests that OK@TA-CeNPs can rapidly scavenge free radicals generated by H_2_O_2_*in vitro*, preventing the corneal epithelial cells from being affected by an oxidative storm, thereby protecting the ocular surface microenvironment.

### 3.6 Targeting reactive oxygen species to ameliorate T cell-mediated inflammation in dry eye syndrome

To investigate the therapeutic effects of OK@TA-CeNPs on a BAC-induced mouse model of dry eye, C57BL/6 mice were administered 5 μL of 0.2% BAC solution twice daily for one week to induce dry eye. On the seventh day, slit-lamp examinations were performed, and fluorescein sodium staining was used to assess corneal epithelial damage. Representative images were captured for clinical scoring, and the mice were divided into four groups based on their corneal clinical scores: Blank, OK, OK@TA, and OK@TA-CeNPs. After another seven days of treatment, corneal clinical scores were reassessed using slit-lamp microscopy on day 14.

**Fig. 5:**
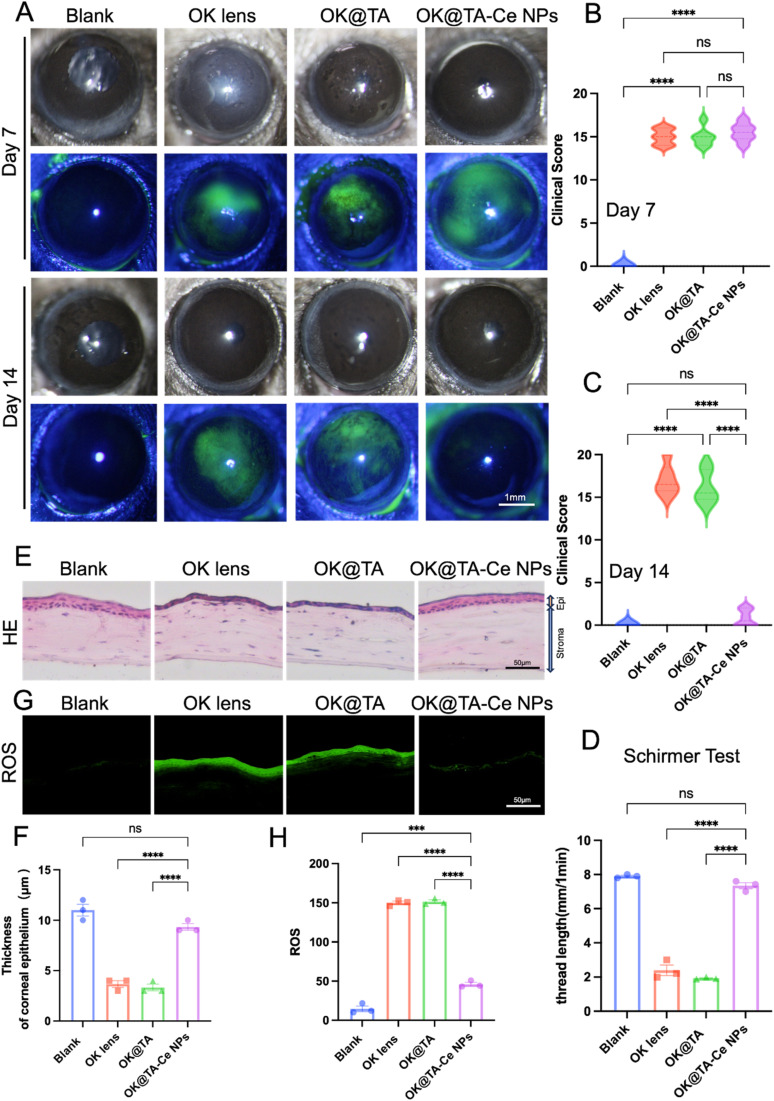
(A) Representative slit-lamp images and fluorescein staining images were collected on days 7 and 14 from each experimental group. (B) and (C) clinical scores of corneal fluorescein sodium staining images were statistically analyzed for each group of mice. (D) Tear secretion levels were measured and analyzed on day 14 for each group. (E) On day 14, histological examination was performed on mouse eyes using frozen sections stained with hematoxylin and eosin. (F) Statistical analysis of corneal epithelial thickness was carried out on day 14. (G) The levels of ROS in the corneal epithelium were evaluated on day 14, and (H) the corresponding statistical analysis was conducted. Results were presented as the mean ± SEM. ***p < 0.001, ****p < 0.0001, and ns stands for not statistically significant. Significance was calculated by one-way analysis of variance.

Representative slit-lamp and fluorescein staining images were captured on days 7 and 14 (Fig. 5A). Clinical scores for corneal fluorescein staining images were statistically analyzed for each group (Fig. 5B and C). The results indicated that compared to the OK group, treatment with TA alone did not achieve the desired therapeutic effect. However, after seven days of treatment with OK@TA-CeNPs, the corneal clinical scores of mice significantly decreased (*N* = 6).

As shown in Fig. 5D, tear secretion was monitored by measuring the length of the phenol red thread in each group on day 14. Statistical analysis revealed that after seven days of treatment with OK@TA-CeNPs, tear secretion in mice had essentially returned to normal levels (phenol red thread length of 7.34 mm), whereas tear secretion in the OK@TA group was significantly reduced (phenol red thread length of 2.39 mm) (*N* = 3).

On day 14, frozen sections of mouse eyeballs from each group were subjected to H&E staining, and representative images were captured (Fig. 5E). The corneal epithelial thickness was statistically analyzed (Fig. 5F). The results indicated that after OK@TA-CeNPs treatment, the corneal epithelium had essentially returned to normal thickness (9.3 μm), while the corneal epithelium in the OK@TA treatment group was significantly thinner (3.6 μm) (*N* = 3).

As shown in Fig. 5G and H, the levels of reactive oxygen species in the corneal epithelium were detected using fluorescence microscopy, and the average relative fluorescence intensity of the ROS probe was statistically analyzed. The results demonstrated that ROS levels were significantly downregulated after OK@TA-CeNPs treatment, with an average relative fluorescence intensity of approximately 46 (*N* = 3).

Encouraged by the previous results, we further investigated the effect of OK@TA-CeNPs on alleviating corneal inflammation in a BAC-induced mouse model of dry eye. After successfully inducing a dry eye model in mice by administering 0.2% BAC solution twice daily for seven days, the mice were randomly divided into four groups based on their corneal clinical scores following fluorescein sodium staining: Blank, OK, OK@TA, and OK@TA-CeNPs. Following seven days of treatment, corneal tissues from each group were collected for frozen sectioning and immunofluorescence staining to monitor the expression of inflammatory cytokines IL-17A and IL-1β.

**Fig. 6:**
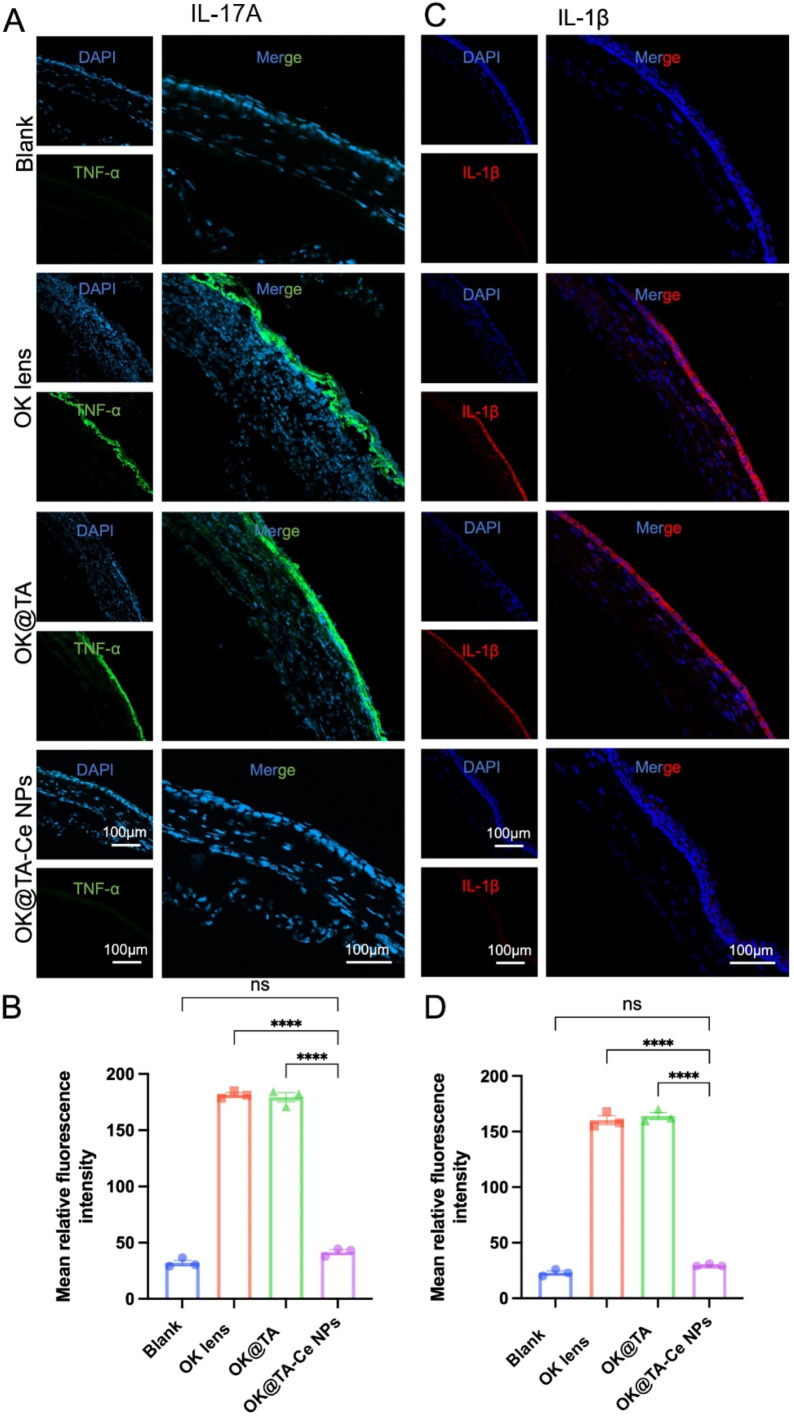
(A) The expression levels of the pro-inflammatory cytokine IL-17A in the corneas of each mouse group were assessed, and (B) the corresponding statistical analyses were conducted. (C) The expression levels of IL-1β in the corneas of each mouse group were examined, and (D) the relevant statistical results were analyzed. Results were presented as the mean ± SEM. ****p < 0.0001, and ns stands for not statistically significant. Significance was calculated by one-way analysis of variance.

As shown in Fig. 6A and B, the expression levels of the pro-inflammatory cytokine IL-17A in the corneas of each group were evaluated, along with the corresponding statistical analysis. The results indicated that the IL-17A expression level was significantly downregulated in the OK@TA-CeNPs treatment group compared to the other groups (Tukey's *q* value of 48.09). In contrast, no significant reduction in IL-17A expression was observed in the OK@TA group (Tukey's *q* value of 1.375) (*N* = 3).

Similarly, Fig. 6C and D show the expression levels of IL-1β in the corneas of each group and the corresponding statistical analysis. The results demonstrated that the IL-1β expression level was significantly decreased in the OK@TA-CeNPs treatment group compared to the other groups (Tukey's *q* value of 50.15). In contrast, no significant reduction in IL-1β expression was observed in the OK@TA group (Tukey's *q* value of 0.8166) (*N* = 3).

## 4 Conclusion

The surface modification of OK lenses with Ce NPs integrated *via* a Schiff base linkage to TA presents a significant innovation in the field of vision correction. This study demonstrates that OK@TA-CeNPs achieve enhanced hydrophilicity, evidenced by a substantial reduction in water contact angle, which is anticipated to alleviate ocular surface discomfort and reduce the risk of dry eye syndrome. The ROS-scavenging capacity of OK@TA-CeNPs, as assessed through methylene blue degradation assays, confirms their ability to neutralize reactive oxygen species effectively. This antioxidant property is crucial for protecting the ocular surface from oxidative stress associated with the use of OK lenses. Cytotoxicity evaluations reveal that OK@TA-CeNPs maintain high biocompatibility with corneal epithelial cells, exhibiting negligible cytotoxic effects. Moreover, the intracellular ROS-scavenging performance of OK@TA-CeNPs, as indicated by diminished ROS fluorescence, suggests a protective role against oxidative damage. In essence, the OK@TA-CeNPs offer a promising strategy for improving patient outcomes in non-surgical myopia correction by combining enhanced wettability, potent antioxidant defense, and biocompatibility. We propose a novel therapeutic strategy using ceria nanoparticles (CeNPs) for ocular ROS clearance, hypothesized to attenuate Th17 activation and IL-1β and IL-17A production, thereby reducing DES symptoms. Looking ahead, future research should focus on the long-term effects of OK@TA-CeNPs on ocular health and the sustainability of their ROS-scavenging properties over extended periods of lens use. Additionally, studies should explore the potential synergistic effects of OK@TA-CeNPs with other ocular therapies to further reduce DES symptoms and inflammation. We predict that the integration of CeNPs into OK lenses could revolutionize the management of DES, especially among OK lens users, by attenuating Th17 activation and the production of IL-1β and IL-17A, thereby reducing DES symptoms. The development of this hybrid coating for OK lenses using Schiff base reactions to link TA with CeNPs aims to neutralize ROS and mitigate inflammation, offering a transformative treatment for DES.

## Ethical statement

All animal procedures were performed in accordance with the Guidelines for Care and Use of Laboratory Animals of Shandong Eye Hospital and approved by the Animal Ethics Committee of Shandong Eye Hospital (SDSYKYY202401-5).

## Data availability

The data supporting this article have been included as part of the ESI.†

## Author contributions

Y. Zheng and H. C. Liu: conceptualization, methodology, data curation, writing – original draft, writing – review & editing. B. K. Lu: conceptualization, methodology. M. C. Dong and X.H. Wang: conceptualization, resources, writing – review & editing, visualization, supervision, project administration, funding acquisition. All authors have read and agreed to the published version of the manuscript.

## Conflicts of interest

The authors declare no commercial conflict of interest.

## Supplementary Material

RA-014-D4RA06759B-s001
