# Supplementary material for: Targeting reactive oxygen species to ameliorate T cell-mediated inflammation in dry eye syndrome: a novel therapeutic approach
Source: RSC Adv. 2024 Nov 18;14(49):36804–15. doi: 10.1039/d4ra06759b (PMC11571118; doi:10.1039/d4ra06759b)
Supplement: RA-014-D4RA06759B-s001 [file RA-014-D4RA06759B-s001.pdf]

Supplementary Information (SI) for RSC Advances.
This journal is © The Royal Society of Chemistry 2024

Targeting Reactive Oxygen Species to Ameliorate T Cell-Mediated Inflammation in Dry Eye Syndrome: A Novel Therapeutic Approach

Yi Zheng[2#], Haochen Liu[2#], Bingkun Lu[2], Muchen Dong[12*], Xinhai Wang[12*]

1, Eye Institute of Shandong First Medical University, Eye Hospital of Shandong First Medical University (Shandong Eye Hospital), Jinan 250021, Shandong, China.

2, School of Ophthalmology, Shandong First Medical University, Jinan 250000, Shandong, China.

#Yi Zheng and Haochen Liu contributed equally to this article.

**Corresponding author:*

***Muchen Dong**, Eye Institute of Shandong First Medical University, Eye Hospital of Shandong First Medical University (Shandong Eye Hospital); School of Ophthalmology, Shandong First Medical University, 372 Jingsi Road, Jinan 250000, Shandong, China.*

Email: dongmuchen0619@163.com

***Xinhai Wang**, Eye Institute of Shandong First Medical University, Eye Hospital of Shandong First Medical University (Shandong Eye Hospital); School of Ophthalmology, Shandong First Medical University, 372 Jingsi Road Huaiyin District, Jinan 250000, Shandong, China.*

Email: mikeannacs@163.com

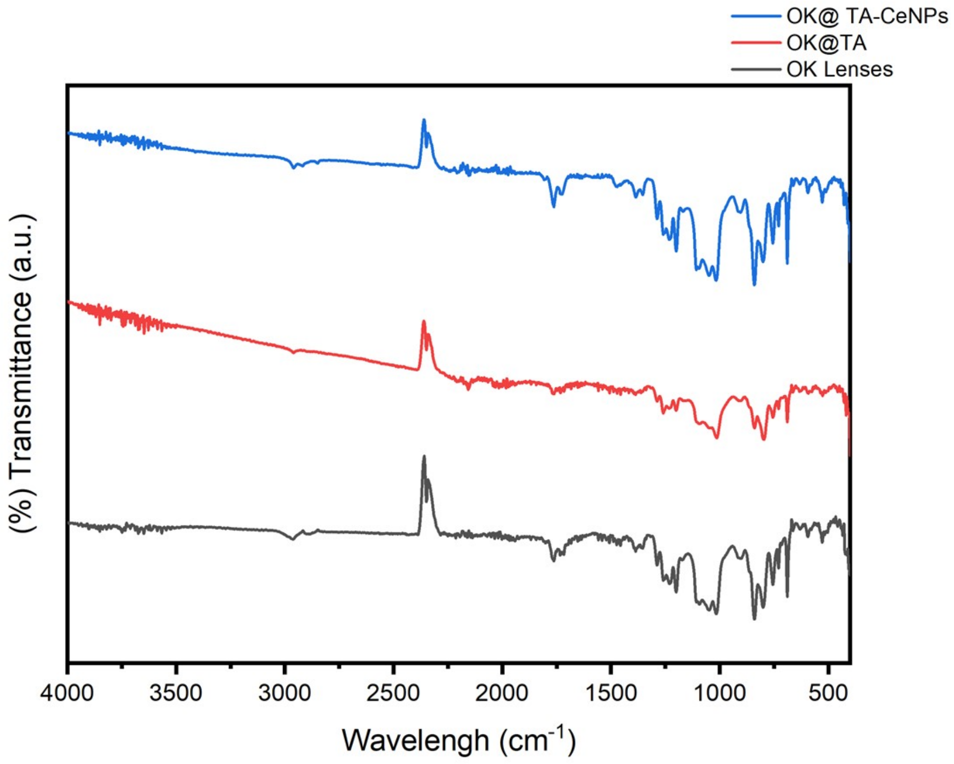

Figure S1. The FTIR analysis of OK, OK@TA, and OK@TA-CeNPs

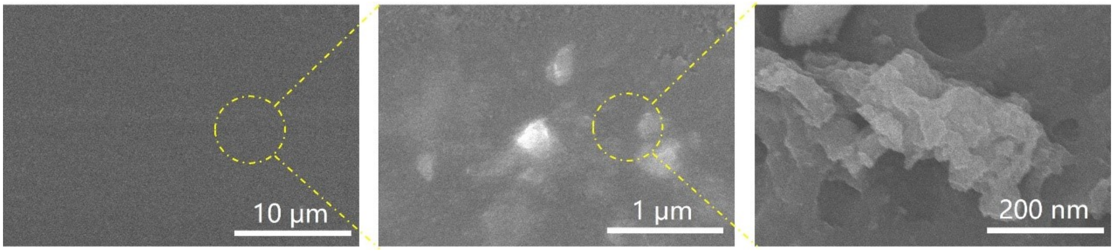

Figure S2. The SEM photo of OK@TA-CeNPs

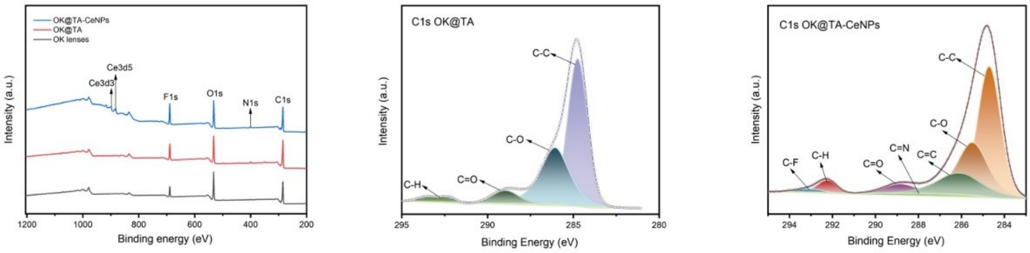

Figure S3. XPS analysis of OK, OK@TA, and OK@TA-CeNPs
